# Digital Gene Expression Profiling to Explore Differentially Expressed Genes Associated with Terpenoid Biosynthesis during Fruit Development in *Litsea* *cubeba*

**DOI:** 10.3390/molecules21091251

**Published:** 2016-09-20

**Authors:** Ming Gao, Liyuan Lin, Yicun Chen, Yangdong Wang

**Affiliations:** 1 State Key Laboratory of Forest Genetics and Tree Breeding, Chinese Academy of Forestry, No.1 Dong Xiaofu, Beijing 10086, China; minggao1984@caf.ac.cn (M.G.); linkeyuanxl@126.com (L.L.); yicun_chen@163.com (Y.C.); 2 Research Institute of Subtropical Forestry, Chinese Academy of Forestry, No.73 Daqiao Road, Hangzhou 311400, Zhejiang Province, China

**Keywords:** terpenoid biosynthesis, digital gene expression, differentially expressed genes, quantitative real-time PCR, *Litseacubeba* (Lour.) Pers.

## Abstract

Mountain pepper (*Litsea* *cubeba* (Lour.) Pers.) (Lauraceae) is an important industrial crop as an ingredient in cosmetics, pesticides, food additives and potential biofuels. These properties are attributed to monoterpenes and sesquiterpenes. However, there is still no integrated model describing differentially expressed genes (DEGs) involved in terpenoid biosynthesis during the fruit development of *L. cubeba*. Here, we performed digital gene expression (DGE) using the Illumina NGS platform to evaluated changes in gene expression during fruit development in *L. cubeba*. DGE generated expression data for approximately 19354 genes. Fruit at 60 days after flowering (DAF) served as the control, and a total of 415, 1255, 449 and 811 up-regulated genes and 505, 1351, 1823 and 1850 down-regulated genes were identified at 75, 90, 105 and 135 DAF, respectively. Pathway analysis revealed 26 genes involved in terpenoid biosynthesis pathways. Three DEGs had continued increasing or declining trends during the fruit development. The quantitative real-time PCR (qRT-PCR) results of five differentially expressed genes were consistent with those obtained from Illumina sequencing. These results provide a comprehensive molecular biology background for research on fruit development, and information that should aid in metabolic engineering to increase the yields of *L. cubeba* essential oil.

## 1. Introduction

Mountain pepper (*Litsea* *cubeba* (Lour.) Persoon) is a fast-growing and aromatic plant of the Lauraceae family, indigenous to Eastern Asia. *L. cubeba* has a diversity of uses. The fruit and bark are commonly used as traditional medicines for the treatment of stomach aches, common cold, inflammation, and coronary heart disease. The aromatic essential oil extracted from the fruits and roots, which possesses an intensely lemon-like odor, has been widely used as a raw material for cosmetics, pesticides, food additives and biodiesel fuel [1]. Meanwhile, the essential oil exhibits a wide-range of bioactivities [2]. These uses of *L.* *cubeba* are attributed to the main components in the fruit oils, monoterpenes and sesquiterpenes, the most dominant component of which is citral, which constitutes more than 80% of the chemical content of the essential oils. Considerable research efforts related to the oil biosynthesis in fruits are urgently needed, and it is believed to be a promising avenue for increasing the content of chemicals of interest.

Citral belongs to the terpenoids. Terpenoids, represented mainly by isoprene (C5), monoterpenes (C10), sesquiterpenes (C15), diterpenes (C20), are the largest category of plant secondary metabolites. They play important roles in plant development, the interactions of plants with the environment, and reproduction through attraction of pollinators and seed disseminators [3]. The biosynthetic pathway of plant terpenoids have been well studied [4,5] and a large number of enzymes are involved this pathway. In the past several years, there has been a significant progress in the identification and characterization of genes encoding the enzymes involved in terpenoid biosynthesis in many plant species, especially in the early steps of biosynthesis. For example, the first plant acetoacetyl-CoA thiolase (AACT) was cloned from radish (*Raphanus sativus*) [6]. 3-Hydroxy-3-methylglutaryl-CoA synthase (HMGS) was identified in *Arabidopsis* [7], *Brassica juncea* [8] and *Hevea brasiliensis* [9]. Three genes encoding 3-hydroxy-3-methylglutaryl-CoA reductase (HMGR) have been identified in snapdragon (*Antirrhinum majus*) [10]. A cDNA encoding a protein with homology to 1-deoxy-d-xylulose 5-phosphate reductoisomerase (DXR) have been identified in tomato [11].

However, there are few studies about the enzymes involved in terpenoid biosynthesis in *L. cubeba*. Chang reported the isolation and functional characterization of three monoterpene synthase (mono-TPS) genes in *L. cubeba* [12]. cDNA encoding DXR was isolated in *L. cubeba*, but functional characterization of the corresponding enzymes was not studied [13]. At the same time, there is still no integrated model describing differentially expressed genes (DEGs) involved in terpenoid biosynthesis during the fruit development of *L. cubeba*. Significant improvements in oil accumulation must be accompanied by changes in the expression of the terpenoids biosynthetic genes during fruit development. Identification of these genes and their regulatory pathways would provide not only new genetic information to understand the development of *L. cubeba* fruit, but also to control gene expression to alter oil accumulation.

In recent years, the rapid development of next-generation sequencing (NGS) technologies has offered new cost-efficient high-throughput approaches for global measurements of gene expression of model or non-model species in recent years [14,15,16]. Digital gene expression (DGE), a tag-based transcriptome sequencing method, can be applied to detect new genes and changes in gene expression with an unbiased view of the transcriptome, greater precision, simpler preprocessing, and consistent results compared to qPCR [17,18]. Furthermore, this technology is useful for estimating the overall gene expression levels at different developmental stages [19,20]. An RNA sequencing project for *L. cubeba* has been carried out using the Illumina platform [2] aiming to promote the further gene expression studies of *L. cubeba.*

In this study, we employed DGE tag profiling using the Illumina NGS platform to analyze the gene expression profiles of five different developmental stages of fruit (60, 75, 90, 105 and 135 days after flowering; DAF) and to identify differentially expressed genes. DGE generated over eight million reads per sample, which produced expression data for approximately 19354 genes. Twenty six candidate genes were selected using pathway analysis and characterized as those responsible for terpenoid biosynthesis. Our results provide a comprehensive understanding of DGE patterns and expression levels during *L. cubeba* fruit development, which should provide an invaluable resource for the identification genes involved in terpenoid biosynthesis, and promote a systematic understanding of the molecular mechanisms of oil production of *L.* *cubeba* in the future. All of the genes identified in our research also provide much information to improve the oil contents and quality of terpenoids in *L. cubeba* breeding*.*

## 2. Results

### 2.1. Accumulation of Citral Concentrations at Different Stages of Fruit Development

**Figure 1:**
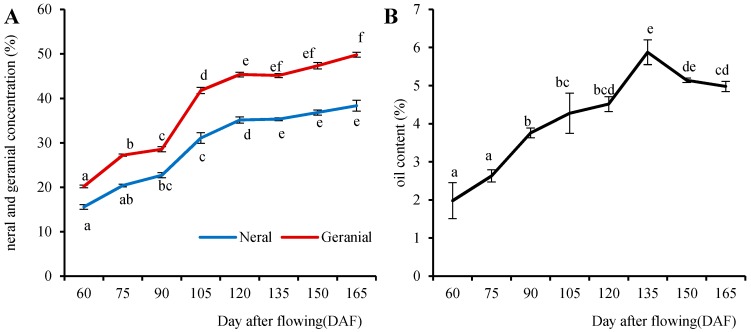
Changes in the concentration of citral and oil content at different developmental stages in *L. cubeba.* Citral concentration (**A**) and oil content (**B**) were measured during the fruit development. The means ± SE are given. The different lower case letters (a–f) indicate significant differences *p* < 0.05). Identical letters indicate the absence of a significant difference and different letters indicate a significant difference.

Because α-citral (geranial) and β-citral (neral) are the major compounds in *L. cubeba*, we analysed the concentration of citral at different stages (60 to 165 DAF) (Figure 1A). To explore oil accumulation during the development of *L. cubeba* fruit, this was also analyzed (Figure 1B). The results indicated that 75 and 90 DAF were developmental stages characterized by rapid increases in the concentration of citral and the oil content, respectively, whereas 105 DAF was the key stage for citral concentration. Furthermore, 135 DAF was the stage when the concentration of citral was stable and the oil content was high. Therefore, to detect the differentially expressed genes with increasing concentrations of citral concentration during the fruit development, the DGE sequencing and qRT-PCR analyses were performed using samples from 60 DAF, 75 DAF, 90 DAF, 105 DAF and 135 DAF stages. 

### 2.2. Analysis of DGE Libraries and Mapping

Five libraries were constructed, and sequence analyses were conducted. Approximately 34.19 million raw reads were generated. Date evaluation of the GC percentage of the clean reads in the five DGE libraries was performed (Table 1). The GC percentages of five libraries range from 46.38 % to 47.57 %. The clean data sets are available at the NCBI SRA with the accession number: SRP073696.

Subsequently, to elucidate the molecular events underlying the DGE profiles, all clean reads were aligned to the reference transcriptome of *L. cubeba*. The average percentage of reads mapped to the reference transcriptome was 89.16% for the five development periods, including mismatching and empty position. Furthermore, after excluding mismatching and empty position, the average percentage of reads mapped to the reference transcriptome was 63.85%. Using the number of reads per kilobase transcriptome per million mapped reads (RPKM) approach, the expression level of each gene was measured by reading the sequencing depth and gene length. The results showed that mRNAs transcribed from the major types of genes were represented by low RPKM values, and only a small proportion of genes was highly expressed (Figure S1). For example, in the 60 DAF stage, the number high expression genes (RPKM > 100) accounted for approximately 1% of the total number of genes (Figure S1-A(1)), whereas the total RPKM values of these genes accounted for 50% of the RPKM values of all the genes (Figure S1-A(2)). In contrast, the number of genes with a lower expression level (RPKM was 0–5) accounted for approximately 60% of the total number of genes, whereas the total RPKM values of these genes accounted for approximately 8% of the RPKM values of all the genes. This expression pattern indicated the non-uniformity and redundancy of mRNA.

**Figure 2:**
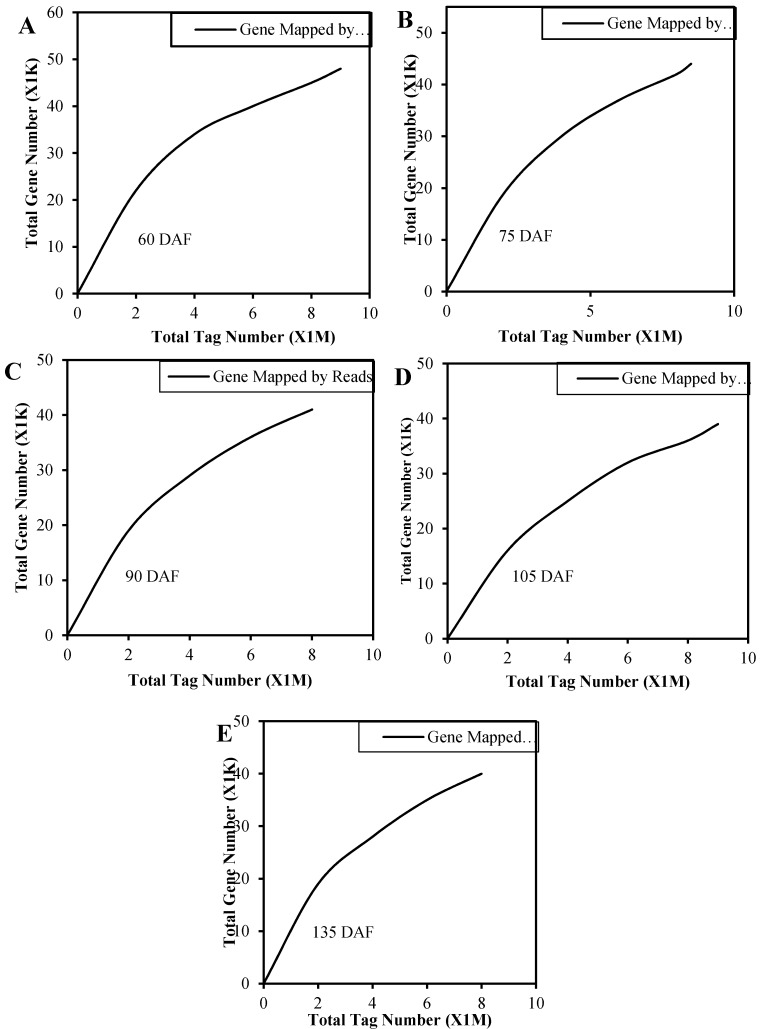
Saturation curve analysis of the digital gene expression tag libraries. (**A**)–(**E**) represents 60 DAF, 75 DAF, 90 DAF, 105 DAF and 135 DAF, respectively. Data are shown as the reads number (X-axis, M represents million) and number of expressed genes (Y-axis, K stands for 1000) obtained using sequencing.

To confirm whether the number of detected genes (RPKM ≥ 1) increased proportionally to the total number of clean reads, sequencing data saturation analysis was performed. Figure 2 shows that the relative number of genes identified increased as the total tag number of tags for all the five libraries. The number of detected genes plateaued when it approached eight million. 

### 2.3. Differential Gene Expression Analysis

**Figure 3:**
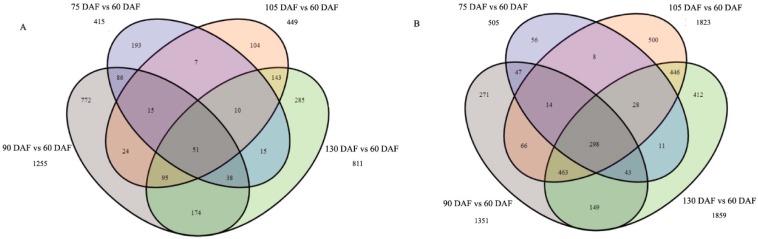
Venn diagram showing all of the differentially expressed genes during development of *L. cubeba* fruit. (**A**) shows all DEGs were the up-regulated in the four contrasts; and (**B**) means all DEGs down-regulated in the four contrasts. The number in one circle represents the numberof stage-specific genes, and the number in two or more intersecting circles represents the number of overlapped genes.

To identify genes that were differentially expressed during the development of *L. cubeba* fruit, the differentially expressed genes (DEGs) between the two samples were identified using statistical criteria (|log_2_ (fold-change)| ≥ 1 and *p*-value ≤ 0.01). Fruit at 60 days after flowering (DAF) served as the control, a total of 920, 2606, 2271 and 2661 genes were differentially expressed at 75, 90, 105 and 135 DAF, respectively. There were 415, 1255, 449 and 811 up-regulated genes and 505, 1351, 1823 and 1850 down-regulated genes identified at 75, 90, 105 and 135 DAF, respectively. The down-regulated genes were more abundant than the up-regulated genes. A Venn diagram showing all of the DEGs is shown in Figure 3. For the up-regulated genes, 51 genes were co-expressed, and 193, 772, 104 and 285 of the up-regulated genes were specific to each of the four respective contrast groups. For the down-regulated genes, 298 genes were co-expressed, and 56, 271, 500 and 412 genes of the down-regulated genes were specific to each of the four respective contrast groups (Figure 3).

### 2.4. GO Functional Enrichment Analysis of the DEGs

**Figure 4:**
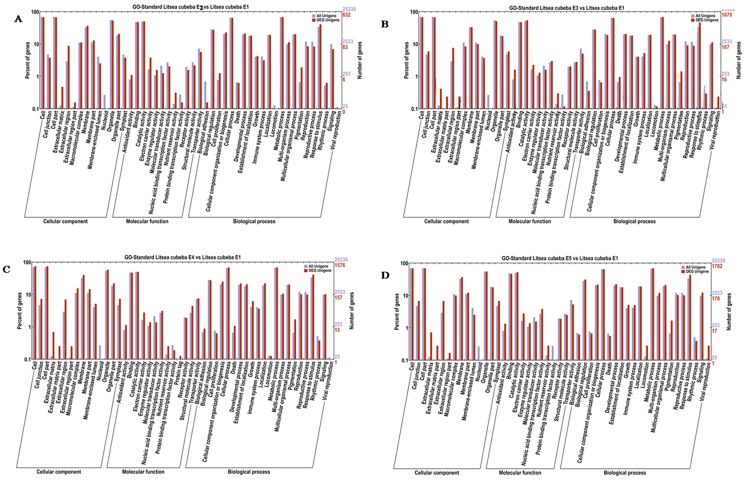
Functional categorization of the differentially expressed genes during development of *L. cubeba* fruit. (**A**)–(**D**) shows the functional categorization of the differentially expressed genes between the 75 DAF, 90 DAF, 105DAF, 135 DAF and 60 DAF (as control) developmental stages.

In our research, DEGs were classified into three functional categories: cellular components, molecular functions, or biological processes (Figure 4). In the 11 subsets (extracellular matrix, extracellular region, extracellular region part, membrane, antioxidant activity, electron carrier activity, enzyme regulator activity, nutrient reservoir activity, developmental process, pigmentation, response to stimulus), ratio of DEG unigenes numbers in all DEG unigenes numbers were more than ratio of unigenes numbers in all DEG unigenes numbers.

Since this study focused on terpenoid biosynthesis, we expected to find that genes associated with this biosynthetic process would be differentially expressed during development of *L. cubeba* fruit. The results showed that 14 subsets of the biological processes category and 20 subsets of the molecular functions category were found to be involved in terpenoid biosynthesis. Furthermore, 75 DEGs were identified to be involved with terpenoid biosynthesis. Within the biological ontology category, the DEGs involved in terpenoid biosynthesis could be further classified into two categories: precursor biosynthetic processes (26 DEGs, two of which were up-regulated and 24 down-regulated) and biosynthetic processes of diverse terpenoids (44 DEGs, two of which were up-regulated and 24 down-regulated). In the molecular function ontology category, 35 DEGs were identified (nine of which were up-regulated and 26 down-regulated) (Table S1).

### 2.5. Pathway Enrichment Analysis of DEGs Using KEGG

KEGG pathway enrichment analysis of the five DEG libraries was performed. All annotated genes were mapped to terms in the KEGG database to search for significantly enriched genes involved in metabolic or signal transduction pathways. A total of 3384 DEGs were mapped to 262 KEGG pathways, including the terpenoid backbone biosynthesis pathways, the ubiquinone and other terpenoid-quinone biosynthesis pathways, the carotenoid biosynthesis pathways, the steroid biosynthesis pathways, the prenyltransferases pathways, the diterpenoid biosynthesis pathways, the limonene and pinene degradation pathways and the geraniol degradation pathways. The 447 DEGs identified in the 60 DAF vs. 75 DAF contrast group were assigned to 120 KEGG pathways. Furthermore, the 1138 DEGs identified in 60 DAF vs. 90 DAF contrast group were mapped to 181 KEGG pathways, the 1320 DEGs identified in the 60 DAF vs. 105 DAF contrast group were mapped to 186 KEGG pathways, and the 1092 DEGs identified in the 60 DAF vs. 135 DAF contrast group were mapped to 180 KEGG pathways. From all those pathways, the terpenoid backbone biosynthesis pathway was selected for further analysis. A total of 26 DEGs were identified in this pathway (Table 2), 14 of which were also identified in the GO analysis. Almost all DEGs (24 of 26) were down-regulated, and only two DEGs (g 8148 and g 56393) were up-regulated (geranylgeranyl diphosphate synthase, type III and geranylgeranyl reductase). In addition, g 4005, g 21893, g 52640, g 5372, g 39745 and g 58660 were up-regulated in the 75 DAF vs. 60 DAF contrast group, g 60046, g 5264, and g 7256 were up-regulated in the 90 DAF vs. 60 DAF contrast group, and g 60046 were up-regulated in the 105 DAF vs. 60 DAF contrast group. Furthermore, g 54221 was up-regulated in all contrast groups except for the 105 DAF vs. 60 DAF contrast group. Figure S2 represents the locations of DEGS involved in the terpenoid backbone biosynthesis pathway. Red or green represents gene was up-regulated or down-regulated genes during the fruit development, respectively.

### 2.6. Clustering of Candidate Genes

**Figure 5:**
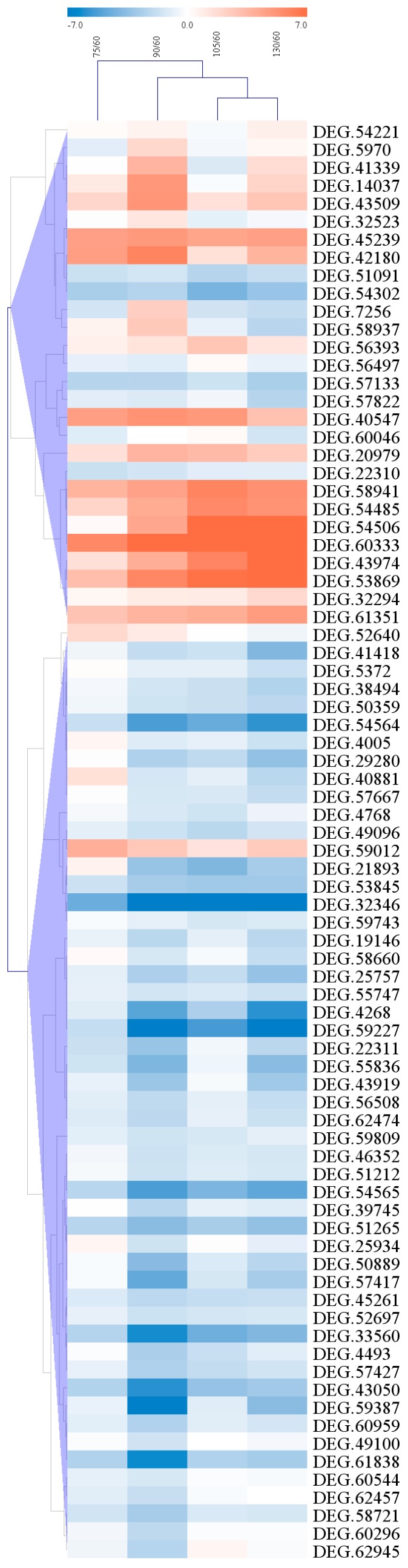
Expression changes and cluster analysis of genes (80 in total) that were differentially expressed between the 75 DAF and 60 DAF (control), 90 DAF and 60 DAF, 105 DAF and 60 DAF, and 135 and 60 DAF contrast groups. The heatmap was generated using Multiple Array Viewer. The rows represent the differentially expressed genes, whereas the columns shows the different contrast groups. Red, blue, and white boxes represent genes showing increased, decreased, or equal expression levels, respectively. Color saturation reflects the magnitude of log2 expression ratio of the RPKM for the four developmental stages and 60 DAF.

The clustering analysis of 80 DEGs (detected by GO and pathway analyses) during the fruit development was performed with the correlated expression profiles. The genes were clustered according to the similarity of their expression patterns. The heatmap of the ratio of the normalized log_2_-transformed RPKM values of the four stages to the 60 DAF RPKM values generated using Multiple Array Viewer (Figure 5). Up-regulated genes are shown in red, while down regulated genes are shown in blue. As shown in Figure 5, the expression level of four DEGs (g 54506, g 60333, g 43974 and g 53869) was all increased during the fruit development, whereas the g 32346, g 54564 and g 59227 showed the opposite trend. Except for these seven DEGs, the expression of others had no unified trend during the the fruit development. Moreover, three DEGs of these seven (g 43974, g 53869 and g 59227) were involved in terpenoid biosynthetic bassed on GO analysis, which may be related the terpenoids biosynthesis and should be investigated in future analysis. 

### 2.7. Confirmation of Differential Genes Using qRT-PCR

**Figure 6:**
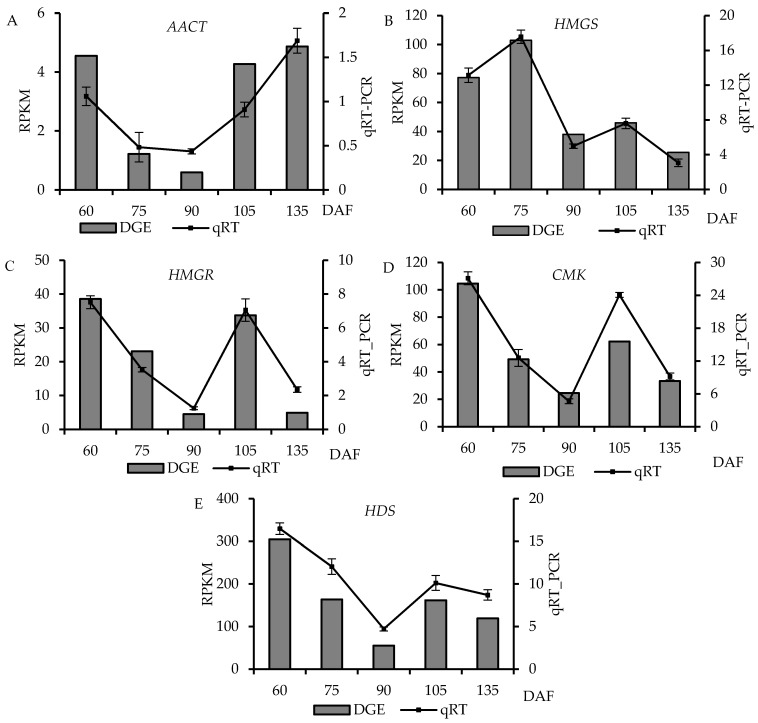
Quantitative RT-PCR confirmation of the differentially expressed genes from DGE analysis. (**A**)–(**E**) represents the differentially expressed genes AACT, HMGS, HMGR, CMK and HDS, respectively. Relative expression levels were calculated using qRT-PCR with UBC as a control. Columns show the means of RPKM values calculated using digital gene expression analysis (Y axis at right). Lines and bars represent the mean and standard error of the relative expression levels (*n* = 3), respectively (Y axis, right). The x-axis indicates the phase of fruit development. RPKM means the reads per kilo base transcriptome per million mapped reads.

To examine the results obtained from the DGE analysis, five genes were selected for qRT-PCR (Table S2). The genes selected for the analysis were involved in two upstream pathways related to terpenoids biosynthesis (mevalonate (MVA) pathway) and methylerythritol 4-phosphate (MEP) pathway). Ubiquitin-conjugating enzyme E2 (UBC) gene served as an internal control gene. The relative expression level of these five genes was compared with the RPKM values from the DGE analysis (Figure 6). The result showed tha the expressed of five genes was consistent between the qRT-PCR and DGE data.

## 3. Discussion

In this study, the DEGs associated with terpenoid biosynthetic processes during *L. cubeba* fruit development were examined using DGE profiling technology. A total of 8459 DEGs were identified across four respective comparisons of developmental stages. Among the identified DEGs, 26 DEGs were associated with terpenoid biosynthesis pathway as identified using pathway analysis. The average percentage of reads mapped to the reference transcriptome was 63.85%. The lack genome sequence date and incomplete transcriptome reads of *L. cubeba* explain the occurrence of the unmapped tags.

Among those differentially expressed genes, more DEGs were down-regulated than up-regulated, and a similar phenomenon was observed in the DEGs involved in terpenoid backbone biosynthesis pathways (24 vs. 2). The fact suggested that these genes may be the negatively controlled genes in terpenoid biosynthesis. However, it didn’t mean lower expression of these genes leaded to lower volatile constituent contents. In other words, the expression levels of terpenoid biosynthetic genes did not correspond with the storage of volatile constituents, since the terpenoids biosynthetic pathway is a complex pathway. Most of the key enzymes involved in this pathway were encoded by multiple genes with different expression patterns and subcellular localizations [21]. Another reason might be plants often transport natural products from a synthesis site to an accumulation site [22]. This phenomenon was verified in *Arabidopsis thaliana*, *Astragalus membranaceus*, and *Tropaeolum majus* [23,24,25].

The biosynthesis pathways of all terpenoids include three main processes, the synthesis of the C5 precursors isopentenyl diphosphate (IPP) and dimethylallyl diphosphate (DMAPP), the synthesis of the immediate diphosphate precursors, and the formation of the diverse terpenoids. A large group of enzymes play a key role in volatile terpene synthesis [4,5]. In this study, we investigated the genes with key roles in the terpenoid biosynthetic process. 

The most predominant chemical constituent in *L. cubebe* essential oil was citral (60–80%), which is an isomeric mixture of geranial and neral [26]. Geranial is produced by geraniol dehydrogenase (GEDH), which catalyses the oxidation of the geraniol [27], and can be transformed into neral by keto-enol tautomerization [28,29]. GEDH was cloned and characterized in a few plant species. Iijima identified two cDNAs encoding NADP+-dependent dehydrogenase that can use geraniol to form citral in *Ocimum basilicum* cv. Sweet Dani, and found that GEDH activity levels were the highest in young leaves which have more glands per unit area [30]. The authors also isolated and characterized a cDNA encoding GEDH in *Zingiber officinale*. The expression levels of ZoGeDH in various ginger plant tissues were in accordance with the accumulation of geranial [27]. In addition, Sato-Masumoto cloned and characterized GEDH in *Perilla* [31]. In our research, although it did not reach the selection criteria for the DEGs (|log_2_ (fold-changed)| < 1), the RPKMs of g36695, which KEGG annotation identified as GEDH, were increased from 60 DAF to 90 DAF, and then decreased until 135 DAF. This trend was inconsistent with the accumulation of citral. It is believed that GEDH expression is decreased during the maturation of fruit and the citral is synthesized during the young stage of fruit development and stored during maturation in *L. cubeba*. This inconsistent phenomenon was also found in *Zingiber officinale* and *Citrus* *unshiu*. In old rhizomes of *Zingiber officinale*, the loss of ZoGeDH expression was concomitant with the accumulation of abundant geranial [27]. In *Citrus unshiu*, the expression of four clones, CitMTSE1, CitMTS3, CitMTS61, and CitMTS62 coding for d-limonene synthase, γ-terpinene synthase, and β-pinene synthase, respectively, appeared mainly in the peel at an early stage of fruit development, whereas they decreased or disappeared in later stages of development [32].

However, the expression level of other genes were consistent with the content. The expression of several compounds, including camphene, caryophyllene, α-pinene, β-pinene, β-myrcene, d-limonene, geraniol (the content were above 1%), were decreased from 60 DAF to 135 DAF (Table S3), which was accompanied by a reduction in RPKM of the related genes during the fruit development (Table S4). The phenomenon was also found in other species. In *Humulus lupulus* L, the expression of *HlSTS1* encoding for the sesquiterpene synthases which catalyse the formation of caryophyllene and humulene was abundant in those tissues with high contents of humulene and caryophyllene [33]. The higher amounts of artemisinin, which is produced by *Artemisia annua*, was mainly a result of higher expression of amorpha-4,11-diene synthase *(ADS)* and artemisinic aldehyde, was a result of higher expression of the amorpha-4,11-diene synthase (ADS) and artemisinic aldehyde Δ11-13 reductase *(DBR2)* genes [34].

In the second phase of terpenoid biosynthesis, IPP and DMAPP are used to produce the immediate precursors of monoterpenes, sesquiterpenes and diterpenes, which are namely geranyl diphosphate (GPP), farnesyl diphosphate (FPP), geranyl geranyl diphosphate (GGPP) by geranyl diphosphate synthase (GPPS), farnesyl diphosphate synthase (FPPS), and geranyl geranyl diphosphate synthase (GGPPS), and the immediate precursors of monoterpenes, sesquiterpenes and diterpenes, respectively. In our research, these three enzymes were found to be differentially expressed during *L. cubebe* fruit development, and most of them were down-regulated. For example, g 5372 (for GPPS) was up-regulated compared to 60 and 75 DAF, but then down-regulated compared to 60 vs. 90 DAF, 60 vs. 105 DAF, and 60 vs. 135 DAF contrast groups. Additionally, g 4493 and g 21893 (for FPPS) and g 50359 and g 57414 (for GGPPS) were down-regulated in four respective groups. These results suggested that the terpenoid biosynthesis might ocurr during the young stage of fruit development and the products stored during maturation. However, the changes in there DEGs were not consistent during the fruit development, which suggests that terpenoid biosynthesis is a complex pathway. For example, GPP exists in both the homodimeric and heterodimeric forms in both angiosperms and gymnosperms [35,36], and two types of subunits constitute heterodimeric GPPS: a large subunit (LSU) and a small subunit (SSU) [37]. These two subunits have different expression parttens for the expression of *GPPS.* In *Salvia miltiorrhiza*, *GPPS.LSU* is less tissue specific compared with that of *GPPS.SSU* and the two types have different gene organization [21]. 

Fourteen enzymes are involved in the first phase of terpenoid biosynthesis, namely AACT HMGS, HMGR, mevalonate kinase (MVK), phosphomevalonate kinase (PMK), and mevalonate diphosphate decarboxylase (MVD), isopentenyl diphosphateisomerase (IDI), 1-deoxy-d-xylulose 5-phosphate synthase (DXS), 1-deoxy-d-xylulose 5-phosphate reductoisomerase (DXR), 2-C-methyl-d-erythritol 4-phosphate cytidylyltransferase (MCT), 4-(cytidine 50-diphospho)-2-C-methyl-d-erythritol kinase (CMK), 2-C-methyl-d-erythritol 2,4-cyclodiphosphate synthase (MDS), (*E*)-4-hydroxy-3-methyl-but-2-enyldiphosphate synthase (HDS), (*E*)-4-hydroxy-3-methyl-but-2-enyl diphosphatereductase (HDR). Studies have suggested that these enzymes could affect the yield of essential oil [38,39]. Therefore, we should focus on expression change of these enzymes. In this research, most genes for these enzymes were detected, and the change trends of those were complicated during the development stage of fruit. Nine of those genea, g 51091 (IDI), g 43919 (HMGR), g 50889 (IDI), g 57667 (HMGS), g 55836 (DXS), g 57427 (DXR), g 62474 (CMK), g 60959 (HDS) and g 56508 (HDR) were all down-regulated in the four respective groups. Furthermore, g 4005 (HMGS) and g 39745 (MDS) was up-regulated in the 60 and 75 DAF contrast group, but then down-regulated in the 60 vs. 90 DAF, 60 vs. 105 DAF, 60 vs. 135 DAF contrast group. However, our results cannot validate a direct relationship between these DEGs and oil accumulation, which should be the subject of further research. 

The terpenoid biosynthetic pathways involve the cooperation of multiple genes. Therefore, it is difficult to increase oil content by overexpressing a single gene. However, in our research, large amount of DEGs involved in the terpenoids biosynthetic pathway were found, which provides a basis to identify key regulatory processes affecting oil accumulation and further molecular genetics and improvement of *L. cubeba*.

## 4. Materials and Methods

### 4.1. Plant Materials

Fruits of *L. cubeba* were collected in 2014 at Fuyang Forest Park, Hangzhou City, Zhejiang Province (Lat. 30°06’ N, 119°96’ E, Alt. 76 m), China. We observed the development process of *L. cubeba* fruit from flowering until fruit maturation from May-August, 2014. Fruits without peduncle were hand-collected at 60 days after flowering (DAF) (the immature stage), and then every 15 days to full maturity, which covered a total range of 105 days. The fruits at every stage were consistently collected from three trees for three replicates. The fruits at every stage were divided into two parts. One part was used to measure the oil contents. The other part was flash frozen in liquid nitrogen and stored at −80 °C until mRNA extraction, sequencing and q-RT PCR validation.

### 4.2. Measurement and Identification of Essential Oil Compositions

To extract the essential oil, fruit hand-collected at 60, 75, 90, 105, 120, 135, 150 and 165 DAF were air-dried and subjected to hydrodistillation using a Clevenger-type apparatus for essential oil. The volatile distilled oils were dried over anhydrous sodium sulphate (Na_2_SO_4_) and stored at 4 °C until analysis. The essential oil compositions were analysed using gas chromatography–mass spectrometry (GC-MS) methodology. GC-MS was performed on an Agilent 6890N gas chromatograph (Palo Alto, CA, USA) equipped with an Agilent 5975B Mass Spectrometer using an HP-5MS fused silica capillary column (30 m × 0.25 mm internal diameter, 0.25 μm film thickness). The temperature program of 50 °C for 2 min was increased to 120 °C for 2 min at a rate of 3 °C/min, then to 250 °C for 2 min at 15 °C/min. The carrier gas was N2 at a flow rate of 1 mL/min. The injected volume was 1.0 μL (1:10 in Et2O) and the injector temperature was 220 °C. Mass spectrometer conditions were as follows: GC-MS interface temperature, 250 °C; ion source temperature, 230 °C; quadrupole temperature, 150 °C; ionisation mode, EI; and ionisation energy, 70 eV. Compounds were identified by comparing their mass spectra with the mass spectra obtained from an MS database (NIST 08). The MS identifications were confirmed by comparing the GC retention times of the analysed samples with those from pure standards. The identification was confirmed by comparing the retention indices (RI) of the samples with those reported in the literature [40,41]. The RI was calculated using a series of n-alkanes (C7–C30) under identical operating conditions. The relative amounts of the individual components were calculated using peak area normalisation.

### 4.3. Total RNA Extraction, cDNA Library Construction, and High Throughput Sequencing

Total RNA was extracted from the fruits collected at 60, 75, 90, 105 and 135 DAF, respectively, using a RN38 EASYspin plus Plant RNA Kit (Aidlab Biotechnologies Co., Ltd., Beijing, China). The yield and quality of RNA samples was determined using a NanoDrop2000 spectrophotometer (Thermo, Wilmington, DE, USA). Approximately 6 µg of total RNA was used for cDNA library construction. The cDNA library was constructed by using a SuperScriptIII 1st Strand cDNA Synthesis Kits (Invitrogen, Carlsbad, CA, USA). Briefly, after mRNA was isolated and purified using Oligo (dT) magnetic beads, it was fragmented using fragmentation buffer. The first-strand cDNA was synthesized using a random hexamers with mRNA as the template. Then the second-strand cDNA was synthesized by adding buffer, dNTPs, RNase H and DNA polymerase I. Double-stranded cDNA was purified using a MinElute PCR Purification Kit (Qiagen, Dusseldorf, Germany), and then repaired by adding EB buffer. The A-tails were ligated to the 3’ ends, and the sequenced joints were added. Agarose gel electrophoresis was used for fragment size selection, and a cDNA library was constructed using PCR amplification. The quality of library was assessed using the Agilent 2100 Bioanalyzer (RNA Nano Chip, Agilent, Cambridge, MA, USA). Finally, the library was constructed and sequenced on the Illumina HiSeq™ 2000 sequencing system.

### 4.4. Sequencing Data and Differentially Expressed Gene Analysis

Raw data (raw reads) in the FASTQ format were transformed into clean data after data-processing: removal of the low quality reads, adaptors and reads containing ploy-N. Then the clean data were used for the subsequent analysis. The quality parameters of clean data including the length, number of reads, and GC-content, were used for the data evaluation. The raw data in the FASTQ format was deposited in the National Center for Biotechnology Information (NCBI) Sequence Read Archive (SRA) database (http://www.ncbi.nlm.nih.gov/sra/?term=). Then the clean data was retained and mapped to the reference transcriptome of *L. cubeba* [2] using BLAT software (http://genome.ucsc.edu/cgi-bin/hgBlat) [42,43]. The reference transcriptome file of *L. cubeba* was downloaded directly from the NCBI website (http://www.ncbi.nlm.nih.gov/). The accession number of the transcriptome is SRA080286. The read numbers mapped to each gene was counted using HTSeq v0.5.4p3 (http://www-huber.embl.de/users/anders/HTSeq/). Then, the expression abundance of the genes was calculated by the number of reads per kilobase of exon model per million mapped reads (RPKM) [44]. Differential expression analysis of two samples was performed using the IDEG6 http://telethon.bio.unipd.it/bioinfo/IDEG6/ [45]. The false discovery rate (FDR) was applied to adjust the P value in multiple tests and statistical analyses. After the P-values were adjusted, the DEGs were obtained by identifying genes with a P ≤ 0.01 and |log_2_ (fold-changed)| ≥ 1.

### 4.5. Functional and Clustering Analysis of the Differentially Expressed Genes 

To annotate, classify, and functionally map the differentially expressed genes, these genes were analysed in the Gene Ontology (GO) and the Kyoto Encyclopedia of Genes and Genomes (KEGG) database using Blast2GO (E-value ≤ 10-5) and BLASTX (E-value ≤ 10-5) programs, respectively. Clustering analysis of differentially expressed genes was conducted using the Multiple Array Viewer (MAV) software (Dana-Farber Cancer Institute, Boston, MA, USA). The heat maps and corresponding HCL tree were constructed using the Pearson correlation method with average linkage. 

### 4.6. Quantitative Real-Time PCR Validation

To verify the data obtained by Illumina sequencing, qRT-PCR was performed on five genes involved in terpenoid synthesis, and the ubiquitin-conjugating enzyme E2 (*UBC*) gene served as an internal control gene [46]. The RNA samples used for the qRT-PCR assays were the same as those used for the DEG experiments. The first-strand c-DNA was synthesized by Superscript III First Strand cDNA Synethesis Kit followed by the RNase H step digestion (Invitrogen). Primer sequences were designed using Primer 3 (http://frodo.wi.mit.edu/primer3/) to amplify products approximately 120–200 bp long. qRT-PCR with the SYBR Premix Ex Taq™ Kit (TliRNaseH Plus) (2X) (TaKaRa, Tokyo, Japan) was carried on 7300 Real Time PCR System (Applied Biosystems, Foster City, CA, USA). According to the manufacturer’s protocol, PCR reactions were run in a 20 µL volumes containing 10 µL of 2× SYBR^®^ Premix Ex Taq™, 0.4 µl of each primer, 0.4 µL of 50 × ROX reference dye, 2 µL of diluted cDNA and 6.8 µl of sterile distilled water. The cycling conditions were 30 s at 95 °C followed by 40 cycles of 5 s at 95 °C and 31 s at 60 °C. Melting curves after 40 PCR cycles were carried out by heating from 60 °C to 95 °C to verify the specificity of each amplicon. Each cDNA was analysed three times. The results were normalized to the expression level of the *UBC*. The relative expression levels of genes were calculated using E^−^^ΔΔCT (gene/UBC)^ [47].

## 5. Conclusions 

In this study, a total 8459 DEGs were identified across four respective comparisons of developmental stages during *L. cubeba* fruit development. Gene ontology analyses identified 75 DEGs associated with terpenoid biosynthesis in *L. cubeba*. Pathway analysis revealed 26 DEGs associated with terpenoid biosynthesis pathways in *L. cubeba*. Our results provide a comprehensive understanding of DEG transcription patterns and expression levels during *L. cubeba* fruit development, particularly the process of oil accumulation. The DEG transcription patterns should provide an invaluable resource for the identification of genes involved in terpenoids biosynthesis in *L. cubeba*, and should promote the systematically understanding of the molecular mechanisms of oil production of *L. cubeba* in the future.

## Figures and Tables

**Table 1:** Summary of DGE sequencing data and mapping results during different fruit development stages of *L. cubeba*.

Samples	Read Number	GC Percentage (%)	Total Mapped Reads ^1^ (Mapped Ratio %)	Uniquely Mapped Reads ^2^ (Mapped Ratio %)
60 DAF	8,971, 349	46.38	7,845,525 (87.45)	5,707,386 (63.62)
75 DAF	8,196,825	47.57	7,349,267 (89.66)	5,410,062 (66.00)
90 DAF	8,531,708	47.26	7,557,873 (88.58)	5,483,701 (64.27)
105 DAF	9,268,559	47.50	8,387,997 (90.50)	5,827,196 (62.87)
130 DAF	8,194,664	47.31	7,346,494 (89.64)	5,122,138 (62.50)

^1^ Total mapped reads: reads mapped to the reference transcriptome, including mismatching and empty positions; ^2^ Uniquely mapped reads: reads mapped to the reference transcriptome, excluding mismatching and empty positions.

**Table 2:** DEGs associated with the terpenoid backbone biosynthesis pathway.

Gene ID	KEGG Annotation	log_2_ Ratio ^1^			
		75 DAF	90 DAF	105 DAF	135 DAF
59743	acetyl-CoA C-acetyltransferase	−0.18057	−0.86507	−1.3505	−1.14886
4005(HMGS)	hydroxymethylglutaryl-CoA synthase	0.405639	−1.05733	−0.74322	−1.62293
57667(HMGS)	hydroxymethylglutaryl-CoA synthase	−0.03242	−1.2824	−1.24393	−1.90689
43919(HMGR)	hydroxymethylglutaryl-CoA reductase (NADPH)	−0.72437	−3.24793	−0.20353	−3.24793
60046(HMGR)	hydroxymethylglutaryl-CoA reductase (NADPH)	−1.01749	0.017278	0.268817	−1.46815
50889(IDI)	isopentenyl-diphosphate delta-isomerase	−0.16506	−3.79442	−1.15056	−2.27085
51091(IDI)	isopentenyl-diphosphate delta-isomerase	−1.77761	−1.41504	−2.58496	−2
55836(DXS)	1-deoxy-d-xylulose-5-phosphate synthase	−1.5009	−3.98089	−0.46063	−3.64386
57427(DXR)	1-deoxy-d-xylulose-5-phosphate reductoisomerase	−0.72763	−2.5119	−1.90923	−1.48543
62474(CMK)	4-diphosphocytidyl-2-C-methyl-d-erythritol kinase	−1.08573	−2.11548	−0.74624	−1.65605
39745(MDS)	2-C-methyl-d-erythritol 2,4-cyclodiphosphate synthase	0.053771	−2.21632	−0.78022	−1.05585
60959(HDS)	(E)-4-hydroxy-3-methylbut-2-enyl-diphosphate synthase	−0.8992	−2.49304	−0.91701	−1.35311
7256(HDR)	4-hydroxy-3-methylbut-2-enyl diphosphate reductase	−1.41504	2.357552	−2	−2
56508(HDR)	4-hydroxy-3-methylbut-2-enyl diphosphate reductase	−0.94617	−2.02185	−0.83504	−1.85798
4493(FPPS)	farnesyl diphosphate synthase	−0.11636	−2.62272	−1.76022	−0.91222
21893(FPPS)	farnesyl diphosphate synthase	0.584963	−11.9658	−11.9658	−11.9658
5372GPPS	geranyl diphosphate synthase	0.131245	−0.93289	−0.93289	−1.80735
36997THS	Alpha-thujene synthase/sabinene synthase	−16.0102	−0.23704	−3.72247	−0.83494
59387THS	Alpha-thujene synthase	−6.97871	−0.70491	−3.65678	−0.9759
50359(GGPPS)	geranylgeranyl diphosphate synthase, type II	−0.42381	−1.53343	−1.6939	−2.1964
52640(GGPPS)	geranylgeranyl diphosphate synthase, type II	2	1.169925	-	−0.41504
54221(GGPPS)	geranylgeranyl diphosphate synthase, type II	0.211504	0.559427	−0.24793	0.752072
57417(GGPPS)	geranylgeranyl diphosphate synthase, type II	−0.17834	−4.77419	−1.30288	−2.77419
8148(GGPPS)	geranylgeranyl diphosphate synthase, type III	13.77314	-	-	-
56393	geranylgeranyl reductase	0.688056	1.369234	2.863498	1.321928
58660	geranylgeranyl reductase	0.259087	−1.28688	−0.2303	−1.84327

^1^ Log_2_ ratio of the 75 DAF, 90 DAF, 105 DAF and 135 DAF RPKM values to the 60 DAF RPKM value.

## Supplementary Materials

Supplementary materials can be accessed at: http://www.mdpi.com/1420-3049/21/9/1251/s1.
