# Supplementary material for: Digital Gene Expression Profiling to Explore Differentially Expressed Genes Associated with Terpenoid Biosynthesis during Fruit Development in Litsea cubeba
Source: Molecules. 2016 Sep 20;21(9):1251. doi: 10.3390/molecules21091251 (PMC6272835; doi:10.3390/molecules21091251)
Supplement: Supplementary file 1 [file molecules-21-01251-s001.pdf]

Supplementary Materials: Digital Gene Expression Profiling to Explore Differentially Expressed Genes Associated with Terpenoid Biosynthesis during Fruit Development in *Litsea cubeba*

Ming Gao, Liyuan Lin, Yicun Chen and Yangdong Wang

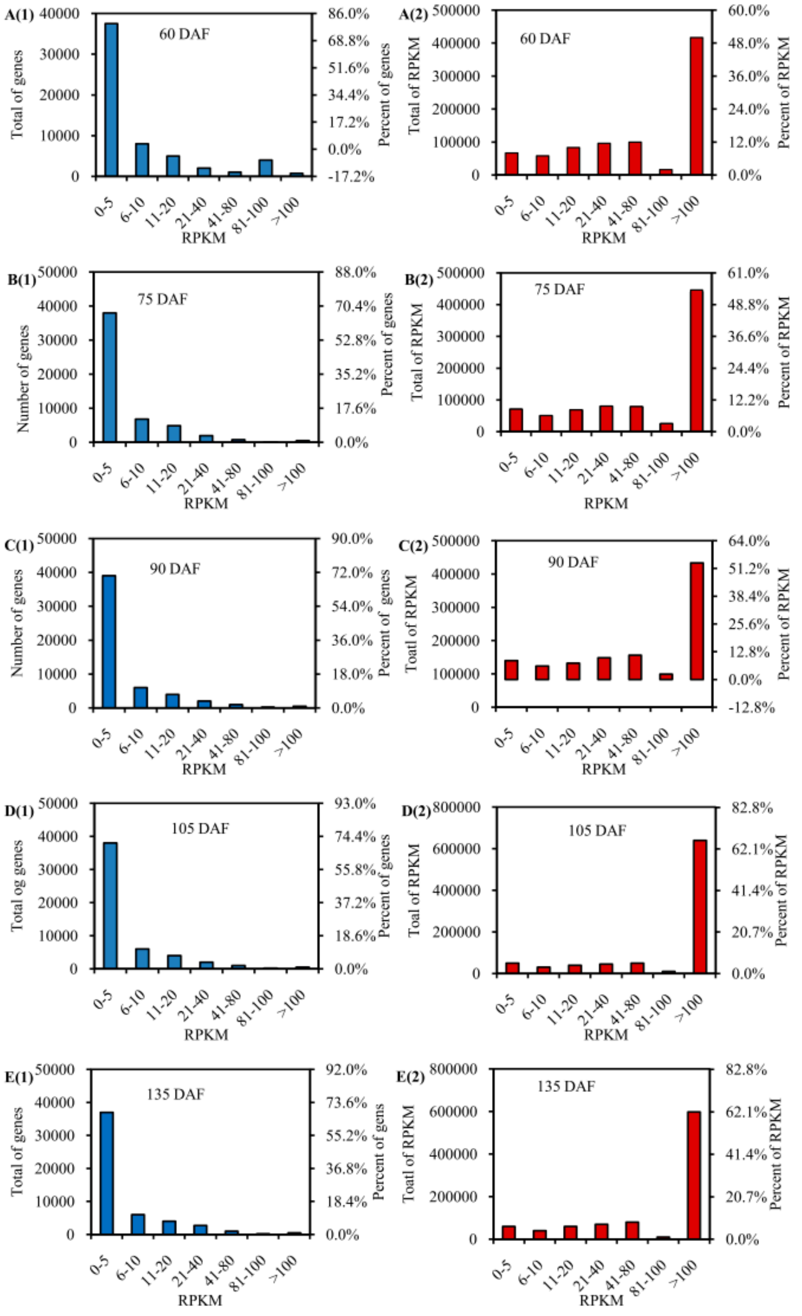

Figure S1. RPKM distribution of all genes. (**A**)–(**E**) represents 60 DAF, 75 DAF, 90 DAF, 105 DAF and 135 DAF, respectively. (1): The x-axis represents the range of RPKMs, whereas the y-axis denotes the number of genes. (2): The x-axis represents the range of RPKMs, whereas the y-axis denotes the total RPKM values of all the genes in this range. RPKM means the reads per kilo base transcriptome per million mapped reads.

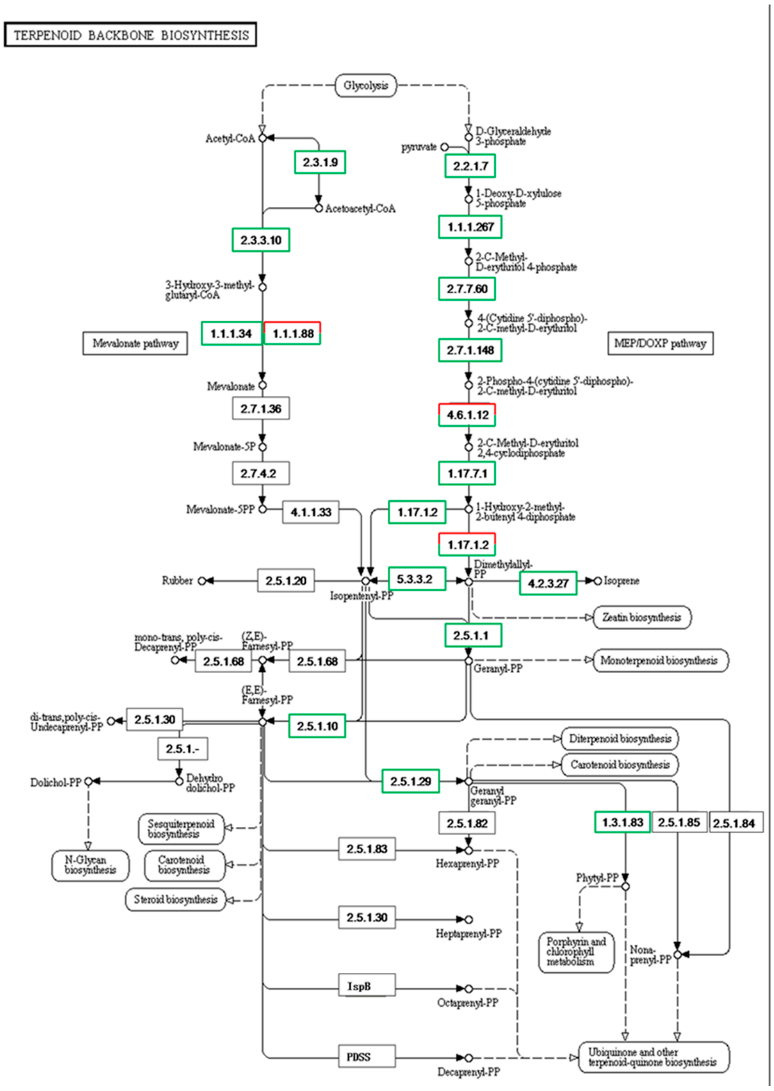

Figure S2. Terpenoid backbone biosynthesis pathway in *L. cubeba*. Red or green rectangles indicate up-regulated or down-regulated genes, respectively.

Table S1. DEGs identity and Gene Ontology annotations.

GO	GO Annotation	Gene ID
Biological Process	monoterpenoid biosynthetic process (GO:0016099)	5970
	terpenoid biosynthetic process (GO:0016114)	62474; 56508; 43974; 34118; 55836; 53869
	isoprenoid biosynthetic process (GO:0008299)	4005; 4493; 43919; 50359; 59387; 57667; 25404; 59227
	pentacyclic triterpenoid biosynthetic process (GO:0019745)	4768; 45261; 19146; 49096; 56497
	diterpene phytoalexin metabolic process (GO:0051501)	50359
	isopentenyl diphosphate biosynthetic process(GO:0019288)	60959; 62474; 49100; 56508; 32294; 7256; 55747; 39745; 52697; 25934; 58721; 38494; 46352; 62457; 4268; 57427; 25757; 51212
	glyceraldehyde-3-phosphate biosynthetic process (GO:0046166);	51212
	coenzyme biosynthetic process (GO:0009108)	60959; 55747; 4268; 57427; 25757;
	coenzyme A metabolic process (GO:0015936)	43919
	aromatic amino acid family metabolic process (GO:0009072)	60959; 4268; 57427; 25757;
	carotenoid biosynthetic process (GO:0016117)	58937; 32294; 60959; 60296; 4268; 57427; 54564; 39745; 60544; 51265; 53845; 25757; 54565
	gibberellin biosynthetic process (GO:0009686)	60333; 61838; 61351; 43050; 22310; 22311; 20979; 32523; 41339; 57133
	regulation of abscisic acid biosynthetic process (GO:0010115)	32347; 32346
	abscisic acid biosynthetic process (GO:0009688)	40547; 54485
Molecular Function	myrcene synthase activity (GO:0050551)	5970
	(R)-limonene synthase activity (GO:0034002)	36997
	terpene synthase activity (GO:0010333)	7718; 59387; 25404; 33560; 59227; 44865
	4-hydroxy-3-methylbut-2-en-1-yl diphosphate synthase activity (GO:0046429)	60959; 7256; 56508
	hydroxymethylglutaryl-CoA synthase activity (GO:0004421)	4005; 57667
	myrcene synthase activity (GO:0050551)	5970
	hydroxymethylglutaryl-CoA reductase (NADPH) activity (GO:0004420)	43919
	1-deoxy-D-xylulose-5-phosphate reductoisomerase activity (GO:0030604)	57427
	4-hydroxy-3-methylbut-2-en-1-yl diphosphate reductase activity (GO:0051745)	56508; 7256
	acetoacetyl-CoA reductase activity (GO:0018454)	62945
	oxidoreductase activity, acting on NADH or NADPH, NAD or NADP as acceptor (GO:0016652)	59809
	oxidoreductase activity, acting on NADH or NADPH, quinone or similar compound as acceptor (GO:0016655)	54302
	oxidoreductase activity, acting on paired donors, with incorporation or reduction of molecular oxygen, NADH or NADPH as one donor, and incorporation of one atom of oxygen (GO:0016709)	43509; 54506; 5294
	NADPH dehydrogenase activity (GO:0003959)	57822
	NADPH binding (GO:0070402)	57427
	carotenoid isomerase activity (GO:0046608)	51265; 60296
	C-19 gibberellin 2-beta-dioxygenase activity (GO:0052634)	38935; 58941
	gibberellin 3-beta-dioxygenase activity (GO:0016707)	38935; 29280
	abscisic acid glucosyltransferase activity (GO:0010294)	41418; 40881; 42180; 14037
	(+)-abscisic acid 8'-hydroxylase activity (GO:0010295)	45239; 59012
	farnesyltranstransferase activity (GO:0004311)	50359

Table S2. Real-time PCR confirmation of differential gene expression.

Name	Forward Primer	Reverse Primer
acetoacetyl-CoA thiolase (AACT)	5′-ACTTCTGCTGACGCTTCGTT-3′	5′-AGCTGGAGCCATGAGATGAT-3′
HMG-CoA synthase (HMGS)	5′-TTCTGAGGAATGCCAGCTCT-3′	5′-TGTTGCCAACTTGTTTTGGT-3′
HMG-CoA reductase (HMGR)	5′-AAAGTTCCCACTGCATCACC-3′	5′-CAAGAGCCTGGAATTTGCTC-3′
4-diphosphocytidyl-2C-methyl-D-e rythritol kinase (CMK)	5′-GGCCTTCAAAGGCTCTCTCT-3′	5′-GGACTCCAGGCACATTTGTT-3′
1-Hydroxy-2-methyl-2-(*E*)-butenyl-4-diphosphate Synthase (HDS)	5′-AATCCGTGTTTCCCTCACAG-3′	5′-GGTGCAAGACTCCTCGGTAG-3′

Table S3. Chemical Composition (%) of the Essential Oil at different stages of fruit development.

Chemistry Content	60 DAF	75 DAF	90 DAF	105 DAF	135 DAF
1R-α-Pinene	3.1	1.7	1.41	0.64	0.53
Camphene	1.61	1.28	0.82	0.38	0.31
β-Pinene	2.81	2.18	1.31	0.68	0.62
β-Myrcene25273	1.98	1.91	1.84	1.28	1.57
d-Limonene	31.64	13.48	13.81	9.31	8.96
Geraniol 22040	3.15	1.64	0.85	0.7	0.63
Caryophyllene	1.29	0.76	1.58	1.85	1.98

Table S4. RPKM change of DEG related with some compounds during the development of *L. cubeba* fruit.

Gene ID	Compounds	$\log_2$ ratio [1]			
		75 DAF	90 DAF	105 DAF	135 DAF
25404	D-Limonene	–2.91	–10.33	-	-
36997	Geraniol	–16.0102	–0.23704	–3.72247	–0.83494
59227	Caryophyllene	–1.86464	–9.12171	–5.38755	–10.1983
59387	β-Myrcene	–0.70294	–6.87505	–0.97369	–3.65008
1025	β-Myrcene	–2.04123	1.270621	–0.55216	–0.19302
57447	β-Myrcene	–1.09638	–1.62044	–0.46047	1.516021
33560	β-Myrcene	–2.38178	–6.22777	–4.37814	–3.86699

[1] $\log_2$ ratio of 75 DAF, 90 DAF, 105 DAF and 135 DAF RPKM value to 60 DAF RPKM value, respectively.
